# Shape representation modulating the effect of motion on visual search performance

**DOI:** 10.1038/s41598-017-14999-1

**Published:** 2017-11-02

**Authors:** Lindong Yang, Ruifeng Yu, Xuelian Lin, Na Liu

**Affiliations:** Department of Industrial Engineering, Tsinghua University, Beijing, 100084 China

## Abstract

The effect of motion on visual search has been extensively investigated, but that of uniform linear motion of display on search performance for tasks with different target–distractor shape representations has been rarely explored. The present study conducted three visual search experiments. In Experiments 1 and 2, participants finished two search tasks that differed in target–distractor shape representations under static and dynamic conditions. Two tasks with clear and blurred stimuli were performed in Experiment 3. The experiments revealed that target–distractor shape representation modulated the effect of motion on visual search performance. For tasks with low target–distractor shape similarity, motion negatively affected search performance, which was consistent with previous studies. However, for tasks with high target–distractor shape similarity, if the target differed from distractors in that a gap with a linear contour was added to the target, and the corresponding part of distractors had a curved contour, motion positively influenced search performance. Motion blur contributed to the performance enhancement under dynamic conditions. The findings are useful for understanding the influence of target–distractor shape representation on dynamic visual search performance when display had uniform linear motion.

## Introduction

Visual search refers to the search for a target among a group of distractors using the eyes^1^. It has received considerable attention in the past decades because many tasks in everyday life and work involve visual search^2–5^. General visual search theories, such as Treisman’s feature integration theory^6^ and Wolfe’s guided search model^7–9^, have been proposed to understand and model the search behavior. These theories mentioned that objects are coded into basic features of different dimensions, such as size, color, and shape, and the feature maps built on those dimensions are integrated into an activation map; attention is guided to items with the highest activation level. Numerous studies on visual search regarding different visual features have been conducted^10^. However, the majority of these studies employ static stimuli although most natural scenes are dynamic, such as the scenes of driving, playing electronic games, and scanning scrolling displays^11–14^.

Motion as a basic feature in visual search should be considered in visual search studies because conclusions drawn from static conditions do not apply in dynamic conditions^15^. Visual acuity under dynamic conditions decreases as velocity increases^16^. Moreover, compared with a static image, a moving image appears less clear as a result of motion blur, which occurs because visual system summates information over time to enhance visual sensitivity^17,18^. Motion results in a camera-like blur and an increase in positional uncertainty relative to static images^19^. Motion blur impairs contrast sensitivity^20^ and the performance of target letter recognition^21^, Vernier discrimination^22^, and spatial interval discrimination^23^. However, motion blur positively affects other visual tasks, such as motion detection^24^ and motion direction discrimination^25^. Motion-induced complexities for dynamic visual search tasks mainly come from four aspects in addition to motion blur. First, motion has various types, such as abrupt onset^26,27^, jumping from location to location^13^, linear motion^28^, and random walk motion^29^. Second, the moving object can be the background^20^, all the target and distractors^11^, only the target^30^, only the distractors^27,31^, or some of the target and distractors^32,33^. Third, the basic components of motion (i.e., speed and direction) may vary for different visual items^34^. Fourth, different search strategies may be adopted under dynamic conditions^11^. Participants in dynamic visual search task may likely adopt passive search or the sit-and-wait strategy^11^, which will result in better performance than active search strategy in certain dynamic environments^35^.

Few studies regarding dynamic visual search have considered the influence of uniform linear motion of display on search performance although it is common in daily life, such as scrolling displays, X-ray security check image flows, and driving scene; in such a motion, all visual items move toward one direction with the same constant speed. Extant studies^36–40^ on uniform linear motion only investigated the influence of velocity on search performance. These studies used different stimuli, but the conclusions are consistent; the performance is worse under dynamic conditions, and large velocity means worse performance. They attributed the performance deterioration to the decrease in visual acuity and the deterioration in visual quality under dynamic conditions. However, none of the studies considered the influence of target–distractor shape representation on search performance.

Shape (or form) is also a basic feature in visual search. However, shape is difficult to define because it is not a unidimensional independent variable, and shape perception is a multifaceted behavior^41^. Various indices, such as circularity, rectangularity, orientation, moments, and curvature, have been proposed to quantify shape properties^42^. These shape features can affect the performance of visual recognition, detection, and search tasks^43,44^. Moreover, shape similarity is often used to describe differences between shapes^5,45,46^. In visual search tasks, shape exerts a modulation effect on performance under static conditions: the influence of the conjunction of tapering and aspect ratio on search rate differs for different targets^47^. For dynamic visual search, studies that focus on the conjunction of motion and shape revealed that search performance differs under different target–distractor shape representations (see, e.g., refs^31,32,48–50^). For example, visual search for a conjunction of movement and shape is efficient if the shape discrimination is easy^51^. However, search efficiency relies on whether the target is moving or stationary if shape discrimination is difficult^52^. The targets in those studies were defined by the conjunction of shape and motion; thus, the relative movement exists between different items. None of them considered the situation of uniform linear movement of display. Whether target–distractor shape representation can modulate the effect of uniform linear motion of display on visual search performance remains unclear.

The first aim of this study was to investigate the modulation effect of target–distractor shape representation on visual search performance under uniform linear motion conditions. In Experiment 1, two visual search tasks (i.e., Experiments 1A and B) with different target–distractor shape representations were performed under static and dynamic conditions. Reaction time under static and dynamic conditions was compared for each task. Shape representation exerted a modulation effect on search performance. The search of targets in Experiment 1A was faster when the stimulus was static than dynamic, whereas the reaction time of Experiment 1B was shorter when the stimulus was dynamic. The second aim was to explore the possible causes of the modulation effect of shape representation. Experiment 2 was designed to confirm the findings of Experiment 1 and investigate search strategy under different conditions. Two search tasks with similar shape settings in Experiment 1 were conducted under static and dynamic conditions. Eye movement data were also recorded and analyzed. Experiment 3 studied the influence of motion blur on search performance. Participants finished two visual search tasks with the same stimuli (i.e., targets and distractors) as those in Experiment 1 under clear and blurred conditions. The stimuli under both conditions were static. The stimuli under clear conditions were exactly the replication of the stimuli from Experiment 1 under static conditions. The same stimuli were processed to simulate the “blurring” of moving objects as stimuli under blurred conditions. Visual scenes are usually complex with many objects^15,35^, and search behavior is different when the set size is large than when it is small^15,26^. However, most visual search studies used a small set size. A large set size was adopted in the present study to overcome the ecological validity problems of previous studies.

## Results

### Experiment 1

**Figure 1:**
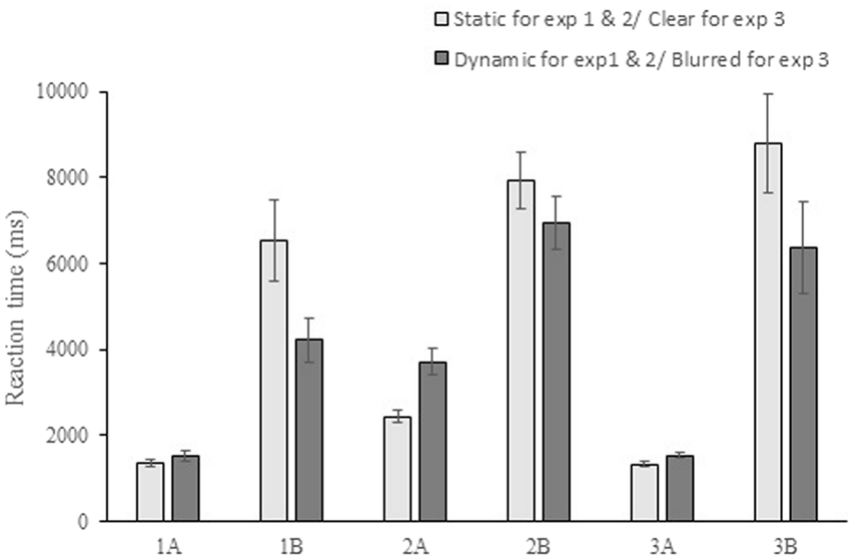
Average reaction time of each visual search task. Error bars show the standard error of the means.

Experiment 1 implemented a 2 × 2 (motion status: static and dynamic; target–distractor shape: O–X for Experiment 1A and Landolt ring–O for Experiment 1B) within-subject experimental design. Each trial had only one target among distractors. Participants were required to search for the target and indicate its location. Thus, false alarm and missing and right reject responses did not exist. False response happened when participants failed to indicate the location of the target. Of all the trials, the proportion of false responses was less than 1%. Thus reaction time rather than accuracy was further analyzed. False responses were excluded from the analysis. Repeated measures ANOVA with motion status and target–distractor shape was performed. The results showed the significant effects on reaction time, i.e., the main effects of motion status (F(1, 18) = 18.01, p < 0.001), target–distractor shape (F(1, 18) = 32.98, p < 0.001), and the interaction of motion status and target–distractor shape (F(1, 18) = 20.41, p < 0.001). In the follow-up paired t-tests, group differences were analyzed. To deal with the family–wise error in multiple comparison, Holm–Bonferroni Method^53^ was used to adjust the *α* level. Compared with that of Experiment 1A, the reaction time (Fig. 1) of Experiment 1B was longer under static (t(18) = −5.60, p < 0.001) and dynamic (t(18) = −5.54, p < 0.001) conditions. Therefore, the effect of target–distractor shape similarity on search performance was robust regardless of motion status; high similarity indicated long search time. In Experiment 1A, participants found the target faster when the stimulus was static than dynamic (t(18) = −3.60, p = 0.002), indicating that motion negatively affected search performance. However, the reaction time of Experiment 1B was shorter when the stimulus was dynamic (t(18) = 4.40, p < 0.001) compared with the static condition, implying that motion enhanced the search performance. The only difference between Experiments 1A and B was the target–distractor shape representations. Thus, the different results of influence of motion were caused by shape representation. The results of Experiment 1 revealed that target–distractor shape representation exerted a modulation effect on search performance when the stimulus was moving.

### Experiment 2

The first aim of Experiment 2 was to confirm the findings of Experiment 1 by use of different stimuli (target–distractor for Experiment 2A: X–P; target–distractor for Experiment 2B: F–P). The target–distractor shape similarity was low for Experiment 2A and high for Experiment 2B. The general Experimental settings and procedure were consistent with those of Experiment 1. The second aim was to investigate whether different dynamic visual search strategies were used for the two shape representations under dynamic conditions, which resulted in the opposite effects of motion on search performance. False responses (less than 1% of all trials) were excluded for the data analysis. Shape representation’s effect on reaction time was significant (F(1, 16) = 60.49, p < 0.001). No significant difference in the reaction time was observed between the static and dynamic conditions (F(1, 16) = 0.41, p = 0.53). The interaction effect of motion status and shape representation on reaction time was significant (F(1, 16) = 22.10, p < 0.001). The reaction time (Fig. 1) of Experiment 2A was shorter than that of Experiment 2B under static and dynamic conditions (static: t(16) = −8.40, p < 0.001; dynamic: t(16) = −5.72, p < 0.001), which was consistent with Experiment 1. The reaction time for Experiment 2A was shorter under static condition than dynamic condition (t(16) = −5.34, p < 0.001), indicating that motion negatively influenced search performance. However, participants had shorter reaction time under dynamic condition than static condition (t(16) = 2.53, p = 0.022) for Experiment 2B, implying that motion positively affected search performance. Thus, the results of Experiment 1 were replicated.

For a single-target visual search task, Chan and Chan^54^ modified the random search model proposed by Morawski *et al*.^55^, as shown in the following equation.

1 $${\rm{ln}}(1-F(t))=a-bt,$$

where *F*(*t*) refers to the cumulative detection probability function, and *t* represents search time.

Equation 1 was used to test whether the participants adopted a random search strategy. The regression results (Table 1) showed that the data fit the model well, suggesting that the search processes can be modeled as random processes.

**Table 1:** Regression results of random search model.

Experimental conditions	Regression equations	R square
Experiment 2A static	${\rm{ln}}(1-F(t))=0.469-0.0006t$	0.988
Experiment 2A dynamic	${\rm{ln}}(1-F(t))=0.082-0.0003t$	0.977
Experiment 2B static	${\rm{ln}}(1-F(t))=0.091-0.0002t$	0.993
Experiment 2B dynamic	${\rm{ln}}(1-F(t))=0.046-0.0001t$	0.991

**Figure 2:**
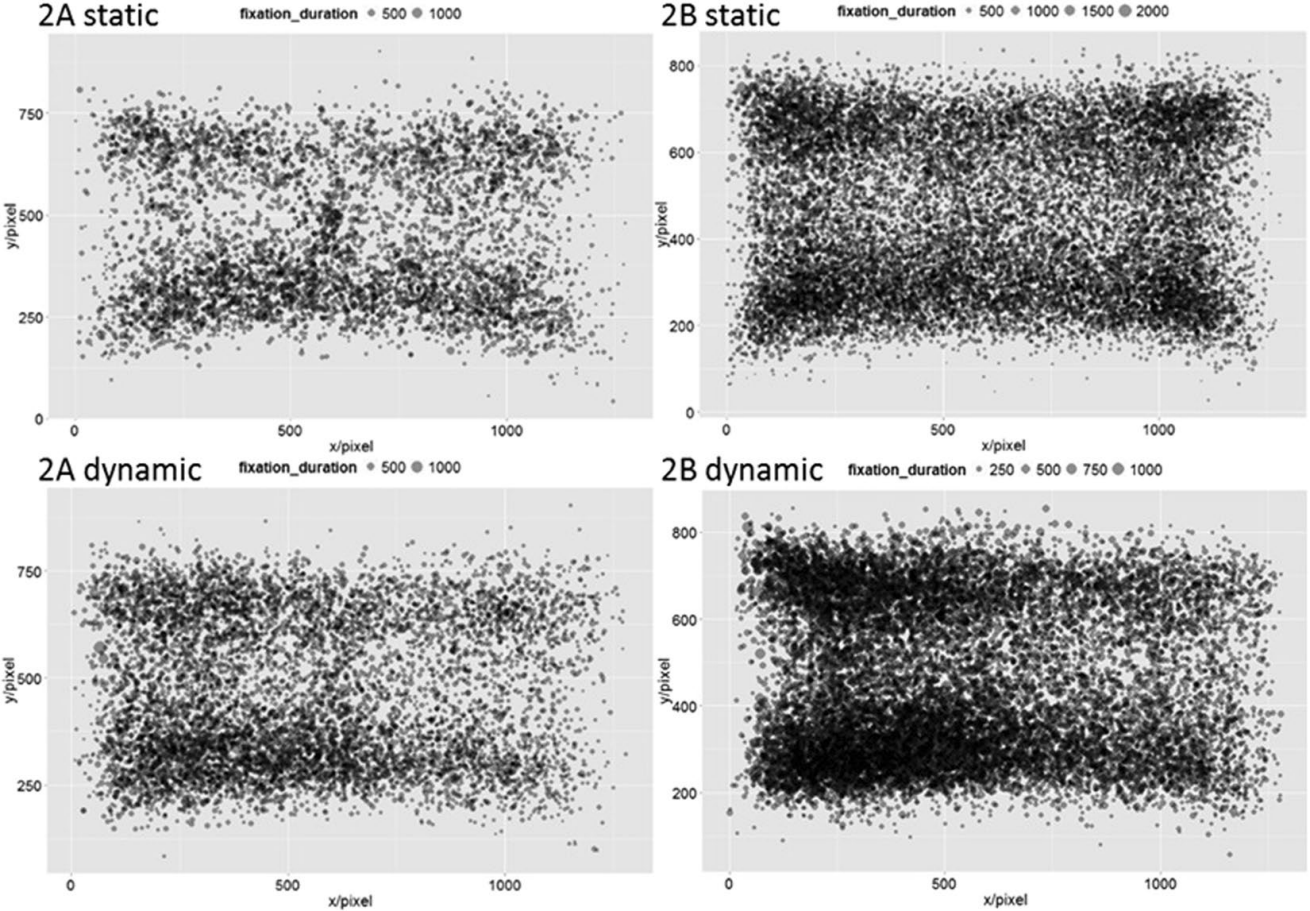
Eye fixations of experiment 2. Each dot is a fixation point. The size of the dots represents the fixation duration (ms).

Figure 2 plots the fixation positions and durations of all participants under different conditions. The passive search strategy results in much smaller fixation number and search area compared with the active search strategy^11,56^. However, Fig. 2 shows that, for both tasks, the fixation number did not decrease and the search area was not reduced when the stimulus was dynamic compared with static conditions. Therefore, search behavior under dynamic conditions was similar to that in static conditions, and participants searched for the target actively for Experiments 2A and B. These results were in accordance with the random search model fitting results, suggesting that the modulation effect of the target–distractor shape representation could not be attributed to different search strategies.

**Figure 3:**
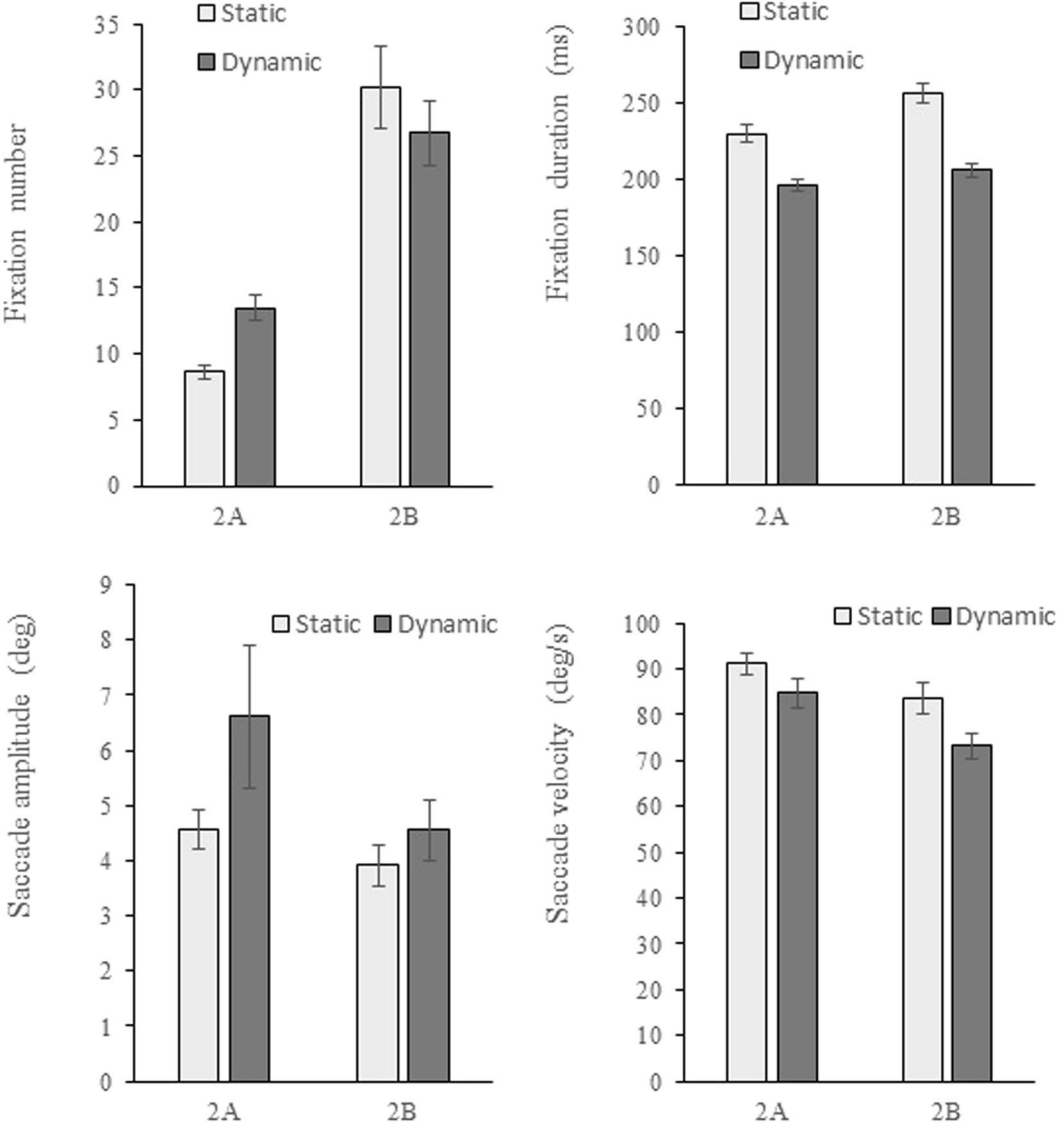
Average fixation number, fixation duration, saccade amplitude and saccade velocity for a single trial in Experiment 2. Error bars show the standard error of the means.

Figure 3 presents eye movement data under different conditions. Under static conditions, 8.6 fixations in average were required to find the target for each trial in Experiment 2A, and over 30 fixations in average were needed for a single trial of Experiment 2B. The mean fixation number of Experiment 2A was significantly smaller than that of Experiment 2B under both conditions (static: t(16) = −7.10, p < 0.001; dynamic: t(16) = −5.20, p < 0.001). Average fixation duration was longer for Experiment 2B than Experiment 2A (static: t(16) = −5.03, p < 0.001; dynamic: t(16) = −3.92, p = 0.001). The saccade amplitude (static: t(16) = 3.93, p = 0.001; dynamic: t(16) = 2.23, p = 0.04) and saccade velocity (static: t(16) = 2.78 p = 0.013; dynamic: t(16) = −6.59, p < 0.001) were shorter for Experiment 2B compared with Experiment 2A regardless of the motion status. Therefore, participants of Experiment 2B had more fixations, longer fixation durations, smaller saccade amplitude, and smaller saccade velocity compared with those of Experiment 2A, and this result was unaffected by motion status. Consequently, search was slower for Experiment 2B than Experiment 2A.

Compared with static conditions, Experiment 2A required more fixations under dynamic conditions and was statistically significant (t(16) = −5.64, p < 0.001). For Experiment 2B, mean fixation number was small when the stimulus was dynamic, but no significant difference was observed contrary to static condition (t(16) = 1.58, p = 0.134). The average fixation durations of Experiments 2A and 2B were shorter when the stimulus was dynamic than static (Experiment 2A: t(16) = 8.33, p < 0.001; Experiment 2B: t(16) = 10.18, p < 0.001). The saccade amplitude of static and dynamic conditions insignificantly differed (Experiment 2A: t(16) = −1.64, p = 0.120; Experiment 2B: t(16) = −1.53, p = 0.145), while the average saccade velocity of dynamic condition was smaller than that of static condition (Experiment 2A: t(16) = 4.75, p < 0.001; Experiment 2B: t(16) = 2.30, p = 0.035). Thus, saccade velocity decreased when the stimulus was dynamic compared with static conditions regardless of the target–distractor shape representation. Therefore, fixation duration and saccade velocity under dynamic conditions decreased regardless of the target–distractor shape representations, unlike those under static conditions. However, the effect of motion on fixation number was modulated by shape representation. For low target–distractor shape similarity, fixation number was large when the stimulus was moving, whereas the effect of motion on fixation number for high shape similarity was insignificant.

### Experiment 3

Experiment 3 was designed to explore the influence of motion blur on search performance. The targets and distractors of Experiments 3A and B were the same as those in Experiments 1A and B. Participants finished search tasks under clear and blurred conditions. The tasks of clear conditions in Experiment 3 were the replication of tasks in Experiment 1 under static conditions. The blurred conditions were used to simulate the dynamic conditions of Experiment 1. False responses (less than 1% of all trials) were excluded from the analysis. Compared with that in Experiment 1, the reaction time of tasks under clear conditions showed no significant difference (Experiment 1A–Experiment 3A: F (1, 35) = 0.05, p = 0.831; Experiment 1B–Experiment 3B: F (1, 35) = 1.19, p = 0.283). The effects of both motion status (F(1, 16) = 10.52, p = 0.005) and shape representation on reaction time were significant (F(1, 16) = 49.77, p < 0.001). The interaction effect of motion status and shape representation was also significant (F(1, 16) = 17.08, p = 0.001). The reaction time of Experiment 3A was shorter than that of Experiment 3B under clear and blurred conditions (clear: t(16) = −6.57, p < 0.001; blurred: t(16) = −4.47, p < 0.001), revealing that the low target–distractor shape similarity resulted in a short reaction time. The search time (Fig. 1) for Experiment 3A was shorter when the stimulus was clear than when it is blurred (t(16) = −4.25, p = 0.001). Participants in Experiment 3B had a shorter reaction time when the stimulus was blurred than when it was clear (t(16) = 3.30, p = 0.004). The results suggested that motion blur negatively affected the reaction time of Experiment 3A whereas positively affected that of Experiment 3B. The influence of motion blur in Experiment 3 was consistent with that of motion in Experiments 1 and 2. Therefore, the inference that motion blur contributed to the performance enhancement of Experiments 1B and 2B was supported.

## Discussion

The present study investigated the modulation effect of target–distractor shape representation on visual search performance when the display had uniform linear movements. Shape representation exerted a strong modulation effect on search performance. If the target–distractor shape similarity was high and the target differed from the distractors in that it had a gap added to the curved shape and the gap had a linear contour and that the corresponding part of distractors had a curved contour, then the influence of motion on search performance was positive. On the contrary, the influence of motion was negative if the target–distractor shape similarity was low. Experiment 2 investigated the search strategies and eye movement behavior of participants using two shape representations under static and dynamic conditions, and found that the modulation effect could not be attributed to search strategies. Experiment 3 compared search performance under clear and blurred conditions and revealed that motion blur contributed to the performance enhancement under dynamic conditions.

The influence of motion on search performance was investigated in Experiment 1 under two target–distractor shape representation conditions. Shape similarity was low for Experiment 1A whereas high for Experiment 1B. Shape similarity exerted a strong effect on search performance; great similarity indicated long reaction time, which was consistent with previous studies^5,46^. Moreover, the present study found that the effect was robust under static and dynamic conditions. As for the influence of uniform linear movement of display on search performance, Experiment 1A had similar results with previous studies; the performance when the stimulus was moving was worse compared with static conditions^36,40^. The form processing system of humans processes moving items with a degraded visual quality due to reduced luminance contrast, retinal smearing, and visual acuity impairment^49,57,58^; thus, the perception of the features of a moving stimulus deteriorates with motion^59,60^. Thus, search time was longer when the stimulus was dynamic compared with static conditions in this study. However, Experiment 1B had a significantly shorter search time under dynamic condition than static condition, which was counterintuitive. No previous dynamic visual search studies have reported similar results. The only difference between the tasks in Experiment 1A and B was the target–distractor shape representation. The contour feature of the target and distractor in Experiment 1A was completely different; thus, the shape similarity was low. However, the target and distractors in Experiment 1B had the same shape except that the target had a gap with a linear contour. The effect of motion on visual search performance was modulated by target–distractor shape representation.

Experiments 2 and 3 were designed to explore the possible causes of counterintuitive results of Experiment 1. Previous studies have shown that subjects may adopt different strategies during visual search^61^. A passive search strategy may result in better performance in certain dynamic environments compared with an active search strategy^11^. The search rate for dynamic search can be faster compared with static conditions if participants adopt a sit-and-wait strategy^56^. Thus, Experiment 2 conducted two search tasks with similar shape settings of Experiment 1 under static and dynamic conditions. In this way, whether different search strategies were used for the two shape representations under dynamic conditions could be determined. Another possible reason was motion blur. Motion blur negatively affects many visual tasks but positively affects some motion detection tasks^62^. Experiment 3 investigated the effect of motion blur on search performance using the same targets and distractors as those in Experiment 1.

The experimental settings of Experiment 2 were consistent with those of Experiment 1, except that different stimuli were used. Following Experiment 1, the target–distractor shape similarity of Experiment 2A was low. The only difference between the target and distractors for Experiment 2B was that a gap with a linear contour existed in the target shape, whereas the corresponding part of the distractor had a curved contour. The results of the reaction time in Experiment 2 confirmed the findings of Experiment 1. Eye movement was also analyzed because of its capability to provide important information of moment-to-moment visual processing, quantitative measures of visual search strategies, and task performance^4,12,63,64^. Under static conditions, participants had to search for the target actively to find the target. However, under dynamic conditions, observers might restrict their attention to certain area to “wait for” the targets, which would result in much smaller search area and much less fixations compared with static conditions. Such dynamic search process was referred to as passive search^11^. In the present study, both search area and fixation times under dynamic conditions were similar with those under static conditions. Thus it supported the conclusion that participants did not adopt passive search strategy under dynamic conditions. The performance enhancement under motion conditions could not be attributed to the search strategy. Humans usually use random strategy when searching for a target in static conditions^65^. This deduction was true even under dynamic conditions.

The influence of target–distractor shape representation and motion on eye movement was also investigated in Experiment 2. The fixation duration was shorter under dynamic conditions compared with that under static conditions. According to the fixate-move model of eye movement control^66^, competition of gaze control in dynamic scenes would produce greater “move” signals, resulting in shorter fixation durations. However, the effect of motion on fixation duration was contrary to studies of Dickinson and Zelinsky^13^, and Smith and Mital^67^. In Dickinson and Zelinsky’s study, the dynamic search task was to find the target in 15 sequentially presented displays in which the target randomly changed their location. The participants adopted the sit-and-wait strategy, resulting in longer fixation duration and much less fixation times than static conditions. However, the participants in the present study used active search strategy. Smith and Mital^65^ found longer fixations in the free viewing tasks of dynamic scenes than those in the free viewing of static scenes. However, the participants in the present study performed search tasks and stimulus had a uniform linear movement, which was different from their study. These different results confirmed the conclusion that gaze behavior difference between free viewing task and search task was large^68^. In short, it can be concluded that eye movement behavior could be different for different visual tasks and under different motion conditions. Saccade amplitudes in Experiment 2 were smaller under dynamic conditions, which was consistent with the results of the visual search in moving radial patterns^61^. Additionally, the influence of motion on fixation number was modulated by target–distractor shape representation. Fixation number will usually increase when the search task is difficult^69,70^. The shape similarity of Experiment 2B was higher than that of Experiment 2A; thus, the task was significantly difficult. Therefore, the mean fixation number of Experiment 2B was high. In addition to fixation duration, saccade amplitude and saccade velocity were small for Experiment 2B, indicating that participants searched slowly and carefully when the task was difficult; this result was unaffected by motion.

Experiment 3 aimed to explore whether motion blur contributed performance enhancement under dynamic conditions in Experiments 1B and 2B. Two search tasks were performed under clear and blurred conditions. The stimuli were the same as those in Experiment 1. Search time under blurred conditions was longer for Experiment 3A and shorter for Experiment 3B compared with that under clear conditions. The results of the blurred task agreed with the results of the first two Experiments under dynamic conditions, indicating that motion blur had effects similar to motion on search performance. For the human visual system, contours are an important source of information about shape^71,72^, and gap exerts a strong effect on Vernier discrimination performance^44^. The only difference between targets and distractors in Experiments 1B and 2B was that a gap with a linear contour existed in the target shape, whereas the corresponding part of the distractors had a curved contour. When the display made a uniform linear movement, the gap and the overall shape was elongated. The target was in a homogenous background because all distractors were the same. The shape difference was amplified because the gap had elongated linear contour, whereas the corresponding part of the distractors had elongated curved contour. Therefore, the target could be easily found. Thus, the performance enhancement caused by motion in Experiments 1B and 2B could be explained by motion blur.

The present study explored the modulation effect of target–distractor shape representation on visual search performance under dynamic conditions. The results indicated four important findings. First, visual search performance might be enhanced or impaired by the uniform linear movement of display, which was modulated by target–distractor shape representation. For search tasks with low target–distractor shape similarity, motion negatively affected search performance. However, for tasks with a high target–distractor shape similarity, the target differed from the distractors in that a gap with a linear contour was added to the target; motion positively influenced search performance because the corresponding part of the distractors had a curved shape. This finding implied that contour feature exerted a mediating effect on dynamic visual search performance when target–distractor shape similarity was high. Second, motion blur contributed to the performance enhancement under dynamic conditions. Third, a random search strategy was adopted regardless of the motion and target–distractor shape representation in the present study. Fourth, high shape similarity indicated large fixation number and duration as well as short saccade velocity and amplitude. Motion negatively influenced fixation duration and saccade velocity, while the effect of motion on fixation number was modulated by target–distractor shape representation. The findings are useful for understanding the influence of shape characteristics of target and distractors on dynamic visual search performance under uniform linear motion conditions.

## Methods

### Experiment 1

#### Participants

Nineteen university students aged 18 to 23 (M = 21.5, SD = 2.9) were recruited for Experiment 1. All the subjects had normal or corrected-to-normal vision. None had relevant knowledge of this study.

#### Stimulus

**Figure 4:**
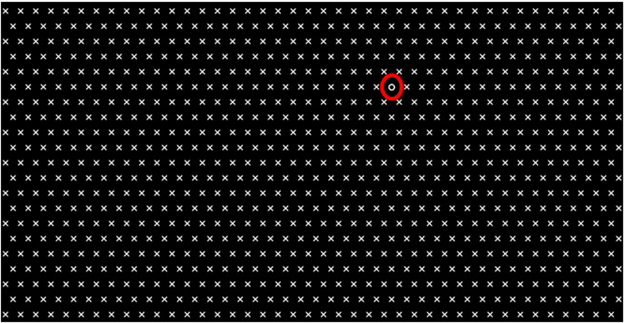
An example of stimulus in Experiment 1A. The distractors are the “X”s and the target is the “O”. The target is marked with an ellipse.

The stimulus image for a single trial contained 860 distractors, and 1 target was uniformly spacing in 21 rows × 41 columns (Fig. 4). The target and distractors were white, and the background was black. Each item was a 30 pixel square. Two visual search tasks were designed in Experiment 1: searching for “O” among “X”s (Experiment 1A) and searching for Landolt ring with a gap oriented in the right among “O”s (Experiment 1B, for example of stimulus refer to supplemental material); the tasks were commonly used in visual search studies^38,43^. The shape of targets and distractors for both tasks differed in contour features. In the first task, the target was a smooth closed curve while the distractor was the cross of two lines. In the second task, the target and the distractor were curves. The difference was that the distractors were closed curves, and the target was an open curve with a gap of a linear contour. The target–distractor shape similarity was low for the first task and high for the second task. For each trial, the target was randomly located in the 3rd–7th and 15th–19th rows and 6th–16th and 24th–34th columns. The participants were required to indicate whether the target was located in the upper or lower half of the screen to prove that the target had been detected. Thus, the targets did not appear near the horizontal dividing line of the stimulus image. The target did not appear in marginal positions. Each Experiment had static and dynamic trials. The stimulus for static trials was stationarily presented. The stimulus for dynamic trials moved horizontally to the right with constant velocity (12 deg/s). The image was presented as a seamless stream such that the items flowed from left to right of the screen, thereby ensuring that all the items, including the target, were always present. The image refreshed every 10 ms to ensure smooth motion^73^.

#### Apparatus

The software was developed using C++. It allowed users to select distractors and the target for different experiments from existing image files. The total rows and columns of the items, the position of the target, and the movement velocity for each trial could be adjusted; these parameters were restored in an input text file. The program was run on a Lenovo Air notebook. The screen was 13.3 inch with a resolution of 1920 × 1080 pixels. The refresh frequency was 50 Hz. The participants were asked to sit on a height-adjustable chair during the experiment. They rested their heads on an adjustable chin rest to limit head movements. The chair and chin rest were adjusted to ensure a distance of 400 mm between the screen and the eyes of the participants. The eyes of the participants were at the same height as the center of the screen, and the distance was adjusted to ensure the angular velocity settings of the stimulus.

#### Procedure

Each participant finished two experiments (i.e., Experiments 1A and B). In Experiment 1A, the experimenter first set the parameters of the software for the experiment, demonstrated how to use the system, and provided some instructions for the participants. The participants then began the practice session. Half of the participants finished 32 static practice trials and then started 40 static experimental trials. They later began the dynamic trials, which included 32 practice trials and 40 experimental trials. The remaining half of the participants performed dynamic trials first and then static trials. For each trial, a white dot was observed at the center of the screen before the exposure of the stimulus image. The participants were required to gaze at the dot and click on it using the mouse. The stimulus image then appeared, and the participants had to search for the target until it was found. The mouse icon was hidden when the stimulus was present. The participants clicked the mouse once the target was found. A response window popped up and asked the participants whether the target was in the upper or lower half of the screen. The participants chose one answer and clicked “OK”; the next trial then began. A message that saying “Test finished” popped up. For static and dynamic trials, the probability that the target was located in each quadrant of the stimulus image was equal (i.e., 10 trials in each quadrant with a random sequence). During practice and experiment trials, the participants were given a break of 2 min after every 10 min of performing the search trials, and they could rest at any time if eye fatigue occurred. Experiment 1B had the same procedure as Experiment 1A.

### Experiment 2

The experimental design of Experiment 2 was consistent with Experiment 1. Two visual search tasks with different targets and distractors were performed under static and dynamic conditions. The targets and distractors for Experiments 2A and 2B were “X” and “P” as well as “F” and “P”, respectively (for example of stimulus refer to supplemental material). Consistent with Experiment 1, the target–distractor shape similarity was low for Experiment 2A and high for Experiment 2B. The only shape difference for the target and distractors of Experiment 2B was that the target had a gap with a linear contour, whereas the distractors had a curve contour of the corresponding part.

#### Participants, Apparatus, and Procedure

Twenty-one university students aged 18 to 24 (M = 22.1, SD = 2.4) participated in Experiment 2. Four participants from Experiment 2 were excluded from analysis due to low eye tracking ratio.

A Dell laptop with a 15 inch screen was used to run the program. The resolution was 1280 × 1080 pixels, and the refresh frequency was 50 Hz. The distance between the screen and the eyes of the participants was 450 mm. The eye movement of the participants was recorded using an SMI iView XTM RED eye tracking system (SensoMotoric Instruments, Teltow, Germany). The sampling rate was 120 Hz, and the spatial resolution was 0.03°. Eye movement data were processed using the SMI BeGazeTM software (SensoMotoric Instruments, Teltow, Germany). Experiments 2A and 2B followed the general procedure of Experiment 1A, except that the 40 experimental trials of each condition were divided into four blocks. Each block started with a five-point calibration to ensure an error of less than 0.5° of visual angle^4^.

### Experiment 3

The task in Experiment 3A was to find the “O” among “X”s under clear and blurred conditions (for example of stimulus refer to supplemental material). The stimuli under both conditions were static. The stimuli of clear conditions were the same as those in the static conditions of Experiment 1A. Under blurred conditions, the same stimuli as clear conditions were processed using Photoshop to simulate the “blurring” effects of moving objects. The blurred stimuli were generated from the original stimuli using the motion blur filter of Adobe Photoshop CC^74^. The task in Experiment 3B was to find the Landolt ring among “O”s under clear and blurred conditions. The other experimental settings of Experiment 3 were consistent with those of Experiment 1.

#### Participants, Apparatus, and Procedure

Seventeen university students aged 19 to 24 (Mean = 21.8, SD = 1.9) were recruited for Experiment 3. The procedure of Experiment 3 was consistent with that of Experiment 1, except that the dynamic trials in Experiment 1 were replaced by blurred trials.

All the experiments were conducted in accordance with the local ethics guidelines and were approved by the Institutional Review Board of Tsinghua University, Beijing, China. The informed consent was obtained from all participants.

### Data Availability

The datasets analyzed during the current study are available from the corresponding author on reasonable request.

## Electronic supplementary material

Supplementary Material
