# Supplementary material for: Shape representation modulating the effect of motion on visual search performance
Source: Sci Rep. 2017 Nov 2;7:14921. doi: 10.1038/s41598-017-14999-1 (PMC5668301; doi:10.1038/s41598-017-14999-1)
Supplement: Supplementary file 1 — Supplementary Material [file 41598_2017_14999_MOESM1_ESM.doc]

**Shape representation modulates the effect of motion on visual search performance**

Lindong Yang^1^, Ruifeng Yu^1, *^, Xuelian Lin^1^, Na Liu^1^

^1^Department of Industrial Engineering, Tsinghua University, Beijing, 100084, China

^*^yurf@tsinghua.edu.cn

**Supplementary Material**

**Additional information of stimulus in each experiment**


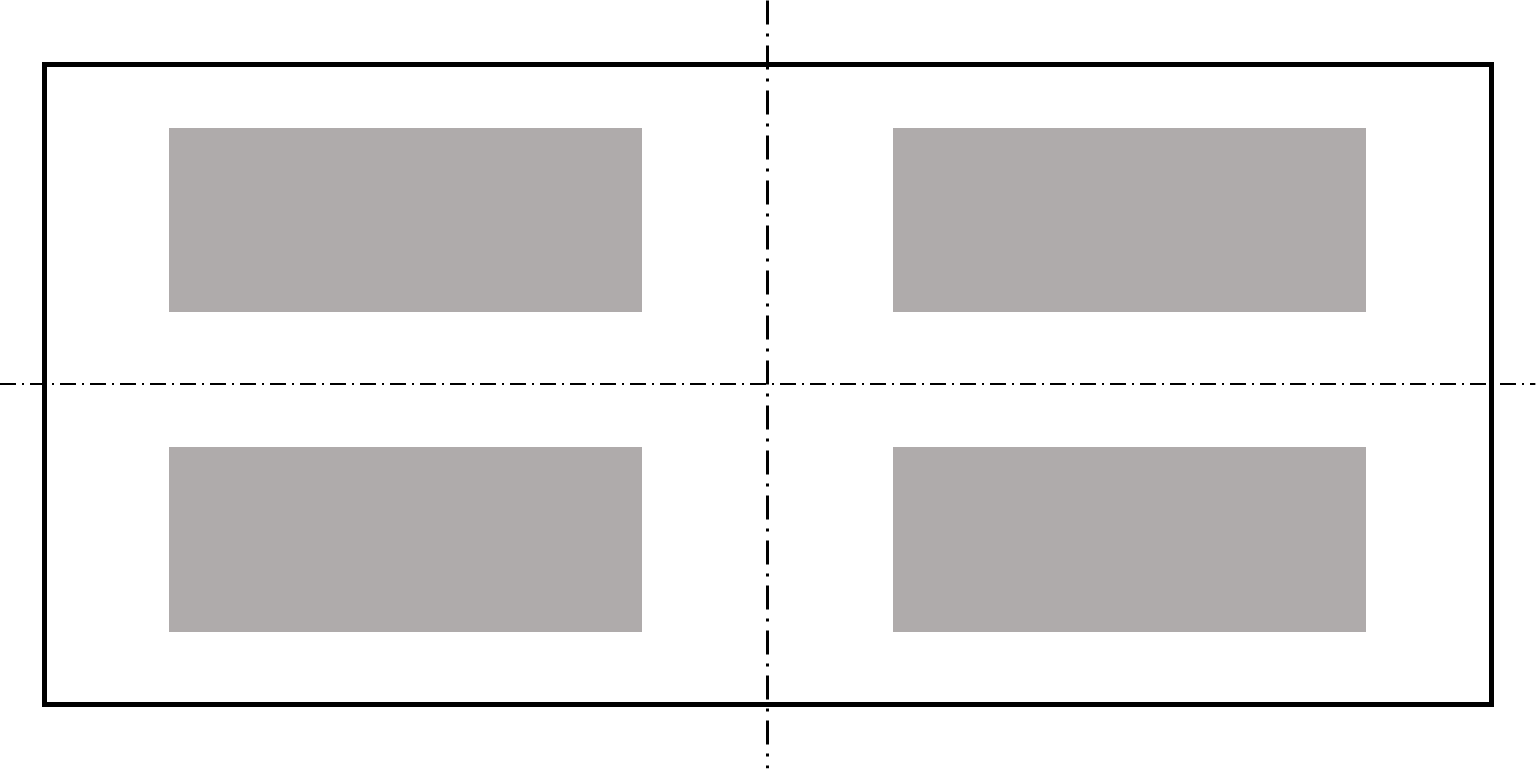


**Figure S1. Target zone of each experiment.** The dash lines are the horizontal and vertical dividing lines of the stimulus area. The dark zones cover all possible positions that the target may locate in.


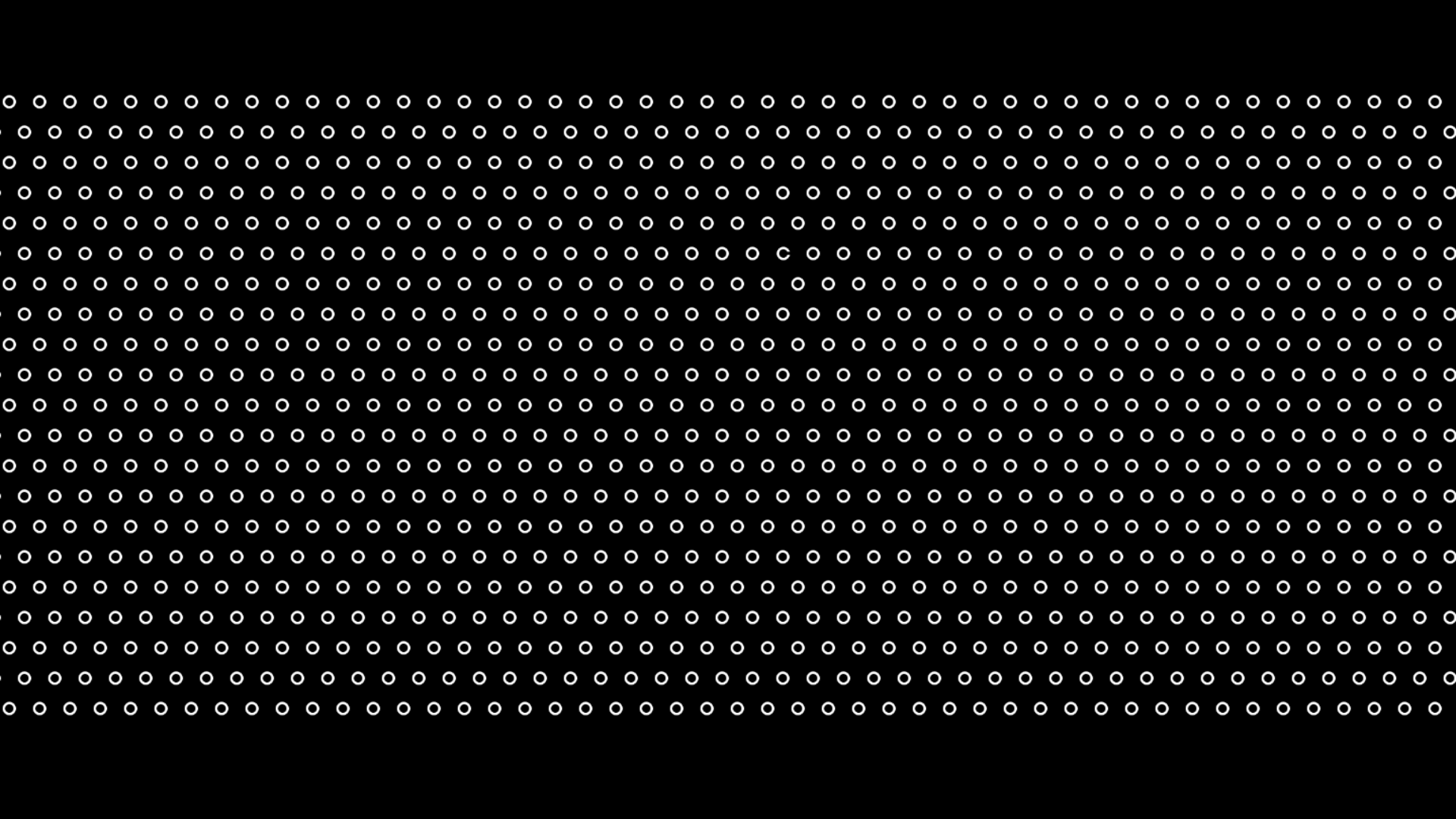


**Figure S2. An example of stimulus in experiment 1B.** The distractors are the “O”s and the target is the Landolt ring with gap oriented in the right. The target is marked with an ellipse.


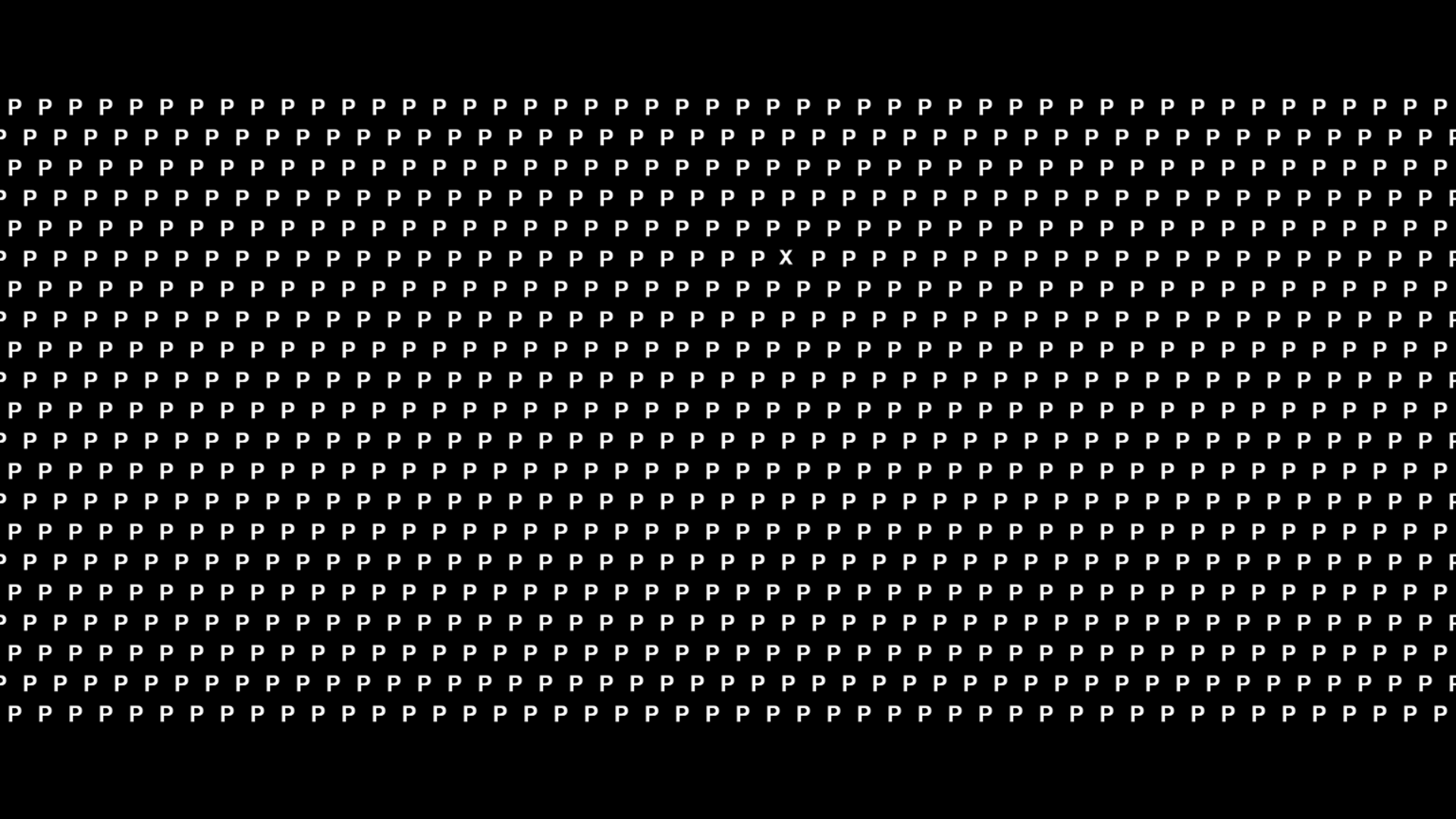


**Figure S3. An example of stimulus in experiment 2A.** The distractors are the “P”s and the target is the “X”. The target is marked with an ellipse.


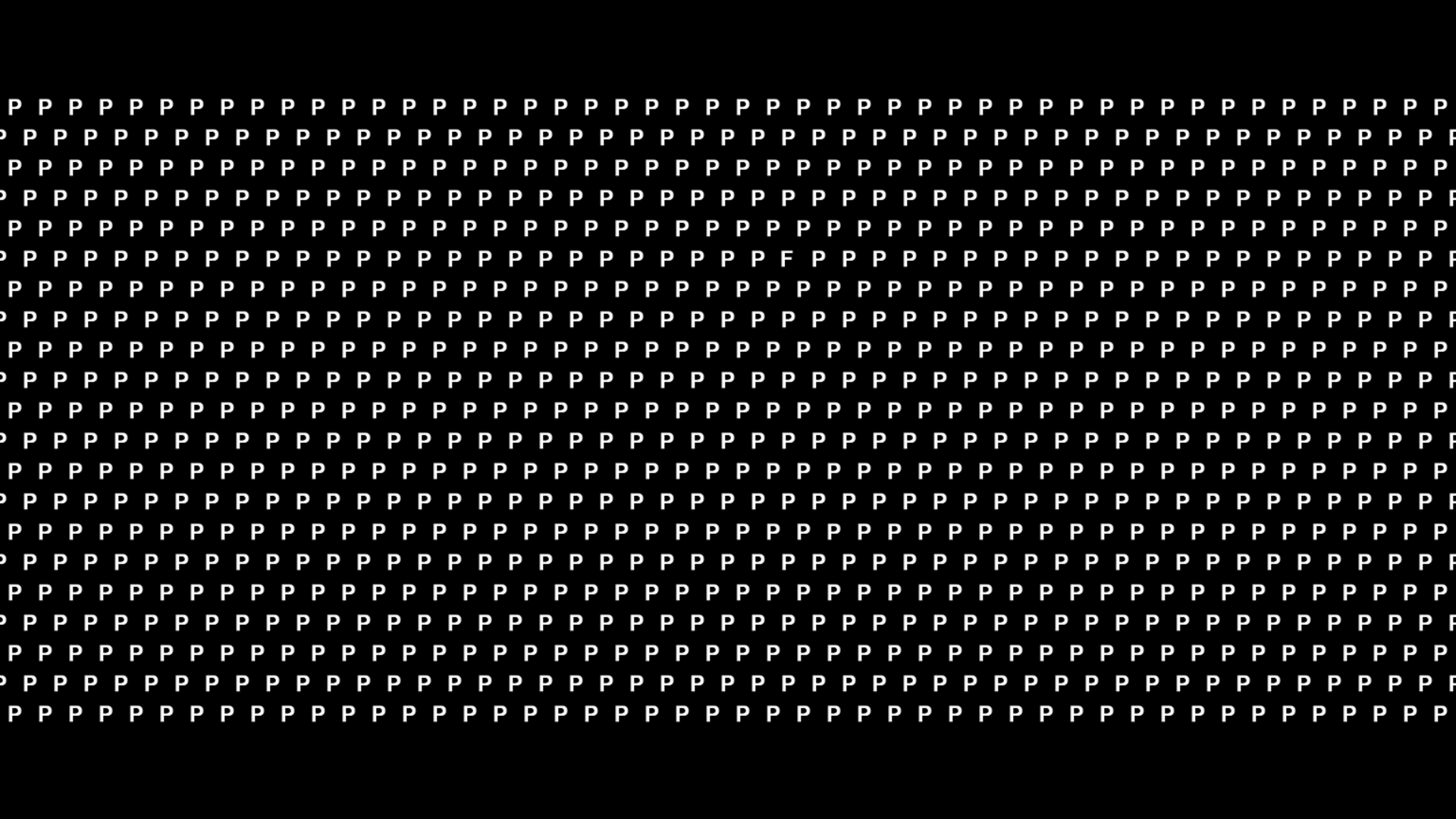


**Figure S4. An example of stimulus in experiment 2B.** The distractors are the “P”s and the target is the “F”. The target is marked with an ellipse.


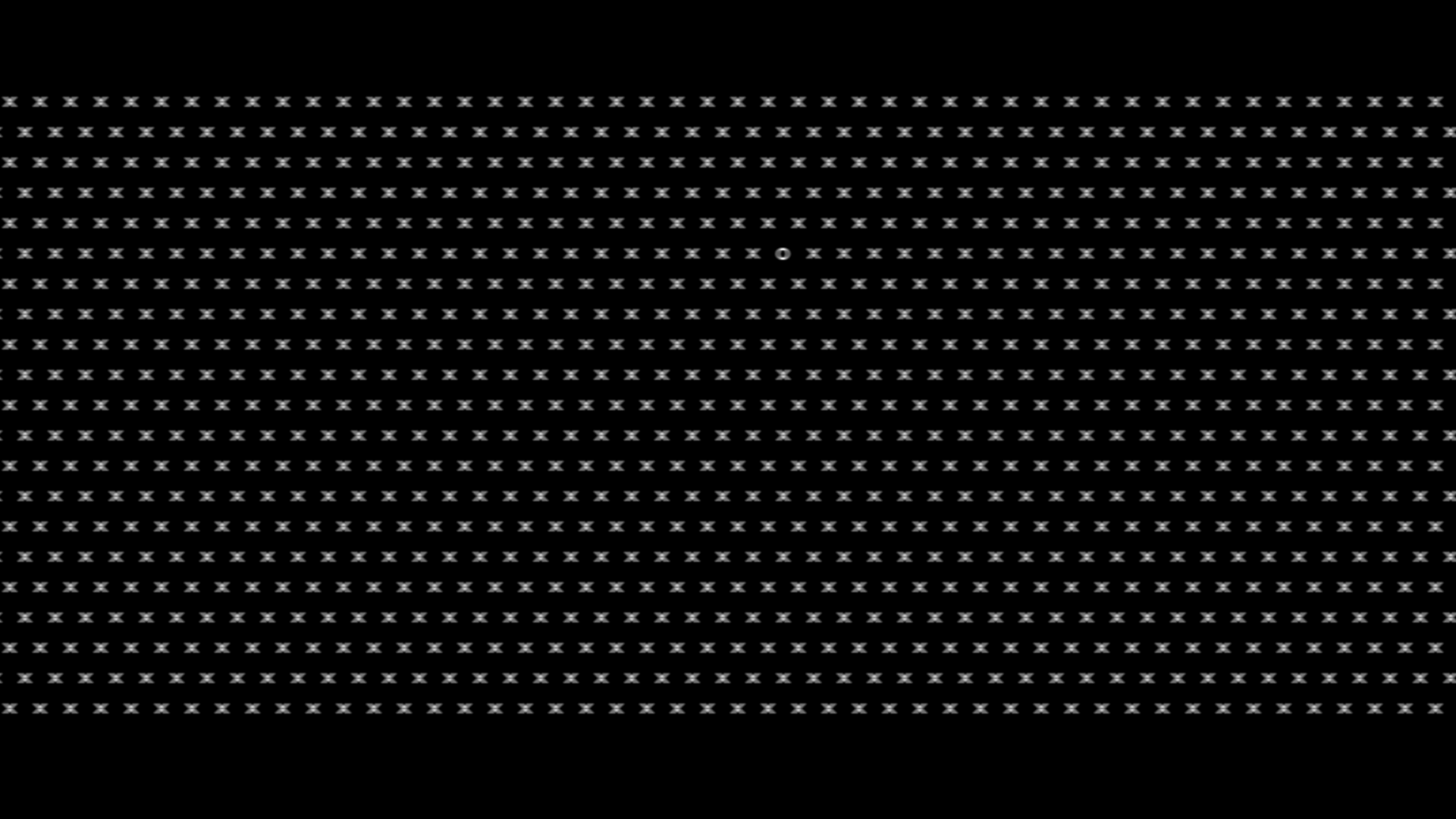


**Figure S5. An example of the blurred stimulus in experiment 3A.** The distractors are the “X”s and the target is the “O”. The target is marked with an ellipse. The stimulus was generated using the motion blur filter of Adobe Photoshop CC. The angle was 0 and the distance was 10 pixels for the filter setting.


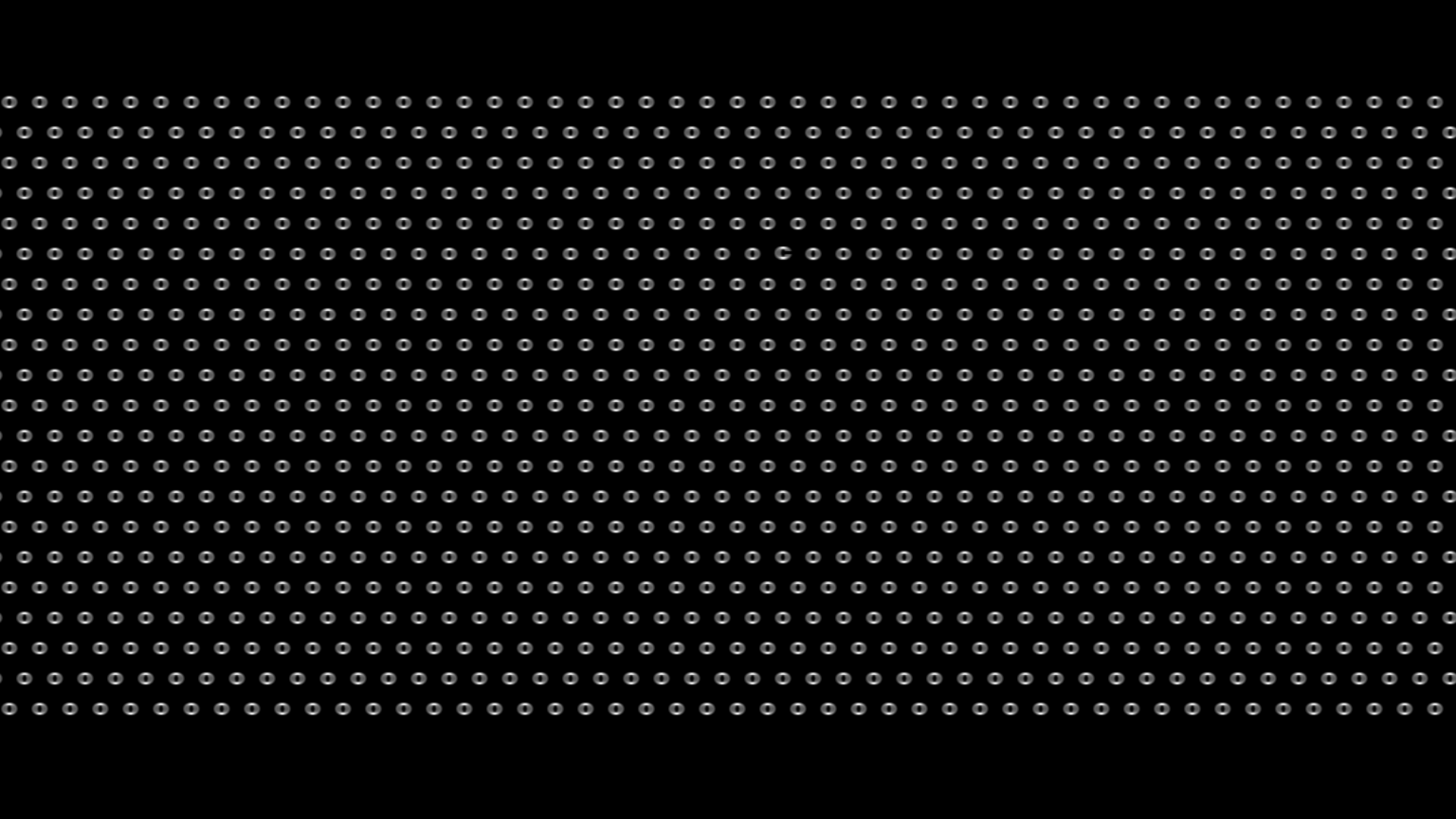


**Figure S6. An example of the blurred stimulus in experiment 3B.** The distractors are the “O”s and the target is the Landolt ring with gap oriented in the right. The target is marked with an ellipse. The stimulus was generated using the motion blur filter of Adobe Photoshop CC. The angle was 0 degree and the distance was 10 pixels for the filter setting.

**Descriptive statistics of experimental results**

| Experiment group | Static/ms | |  | Dynamic/ms | |
| --- | --- | --- | --- | --- | --- |
|  | Mean | SEM |  | Mean | SEM |
| 1A | 1351 | 97 |  | 1510 | 122 |
| 1B | 6535 | 941 |  | 4212 | 508 |
| 2A | 2431 | 144 |  | 3712 | 306 |
| 2B | 7933 | 677 |  | 6933 | 616 |
| 3A | 1326 | 54 |  | 1537 | 68 |
| 3B | 8805 | 1141 |  | 6359 | 1066 |

**Table S1. Means and standard error of means for reaction time of each experiment.**

| Eye movement index | Experiment group | Static | |  | Dynamic | |
| --- | --- | --- | --- | --- | --- | --- |
|  |  | Mean | SEM |  | Mean | SEM |
| Fixation number | 2A | 8.6 | 0.5 |  | 13.5 | 0.9 |
|  | 2B | 30.2 | 3.1 |  | 26.7 | 2.5 |
| Fixation duration  (ms) | 2A | 230.5 | 5.3 |  | 196.7 | 4.1 |
|  | 2B | 256.8 | 6.7 |  | 206.5 | 4.7 |
| Saccade amplitude (degree) | 2A | 4.57 | 0.36 |  | 6.61 | 1.29 |
|  | 2B | 3.91 | 0.36 |  | 4.56 | 0.55 |
| Saccade velocity (degree/s) | 2A | 91.12 | 2.26 |  | 84.71 | 3.03 |
|  | 2B | 83.62 | 3.49 |  | 73.28 | 2.83 |

**Table S2. Means and standard error of means for eye movement indices of experiment 2.**
